# Multifunctional PEG Carrier by Chemoenzymatic Synthesis for Drug Delivery Systems: In Memory of Professor Andrzej Dworak

**DOI:** 10.3390/polym14142900

**Published:** 2022-07-16

**Authors:** Judit E. Puskas, Gayatri Shrikhande, Eniko Krisch, Kristof Molnar

**Affiliations:** 1 Department of Food, Agricultural and Biological Engineering, College of Food, Agricultural, and Environmental Sciences, The Ohio State University, 222 FABE, 1680 Madison Avenue, Wooster, OH 44691, USA; molnarnekrisch.1@osu.edu (E.K.); molnar.182@osu.edu (K.M.); 2 Dantari, Inc., 1290 Rancho Conejo Blvd, Suite 103, Thousand Oaks, CA 91320, USA; gayatri.shrikhande20@gmail.com

**Keywords:** enzyme catalysis, discrete poly(ethylene glycol) dPEG, polymer drug conjugate, modular assembly, doxorubicin, folic acid, Michael addition

## Abstract

This paper describes the synthesis and characterization of new bivalent folate-targeted PEGylated doxorubicin (FA_2_-dPEG-DOX_2_) made by modular chemo-enzymatic processes using *Candida antarctica* lipase B (CALB) as a biocatalyst. Unique features are the use of monodisperse PEG (dPEG) and the synthesis of thiol-functionalized folic acid yielding exclusive γ-conjugation of folic acid (FA) to dPEG. The polymer-based drug conjugate is built up by a series of transesterification and Michael addition reactions all catalyzed be CALB. In comparison with other methods in the literature, the modular approach with enzyme catalysis leads to selectivity, full conversion and high yield, and no transition metal catalyst residues. The intermediate product with four acrylate groups is an excellent platform for Michael-addition-type reactions for a wide variety of biologically active molecules. The chemical structures were confirmed by nuclear magnetic resonance spectroscopy (NMR). Flow cytometry analysis showed that, at 10 µM concentration, both free DOX and FA_2_-dPEG-DOX_2_ were taken up by 99.9% of triple-negative breast cancer cells in 2 h. Fluorescence was detected for 5 days after injecting compound IV into mice. Preliminary results showed that intra-tumoral injection seemed to delay tumor growth more than intravenous delivery.

## 1. Introduction

**Scheme 1:**
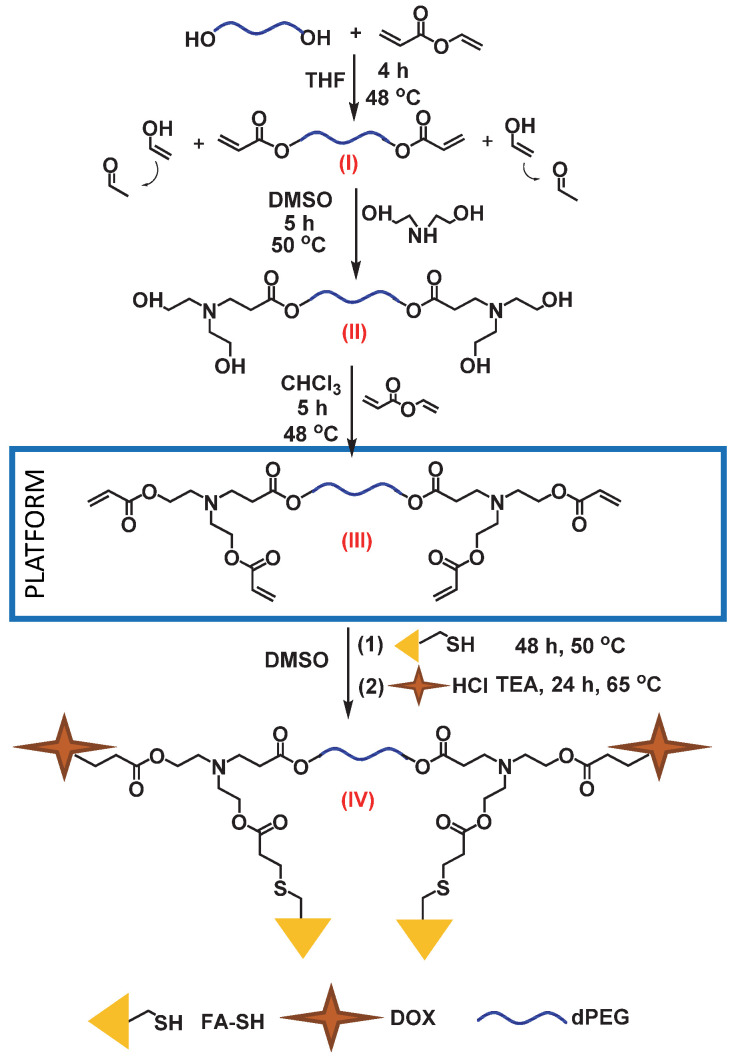
Synthesis of FA_2_-dPEG-DOX_2_: Thiol-functionalized folic acid (FA-SH), Doxorubicin (DOX), discrete poly(ethylene glycol) (dPEG).

Targeted drug delivery systems promise to send cancer drugs to diseased cells without affecting healthy cells, thereby reducing cytotoxicity and minimizing devastating side-effects [1,2,3,4,5,6]. Such delivery systems consist of a drug or diagnostic agent (or both), a linker, a cleavable bond for the release of the drug and a targeting agent, all built into one molecule [7]. Diagnostic and therapeutic agents are being developed that target vitamin receptors (e.g., folate or biotin receptors) that are highly concentrated on the surface of cancer cells [8,9]. Most reports discuss compounds containing folic acid (FA) targeting folate receptors (FR) [10,11]. The two major groups of compounds studied are small molecule drug conjugates and polymeric drug conjugates (PDCs) [12,13]. This latter group showed promise due to increased water solubility and circulation time in the body and multivalent attachment to FRs [14,15,16,17]. However, as a recent review pointed out, the greatest challenge is the inherent heterogeneity of PDCs, coupled with uncontrolled conjugation of diagnostic and therapeutic agents, resulting in polydisperse polymer mixtures [6]. There are a wide variety of polymers used in the synthesis of PDCs with poly(amido amine) dendrimers and poly(ethylene glycol) (PEG) being the most well-known [18,19,20]. We investigated monodisperse PDCs based on PEG, specifically, dPEG (discrete PEG with Đ = 1). First, we synthesized fluorescein (FL)-labeled PEGs containing two FA (FA-FL-PEG-FL-FA, in comparison with compounds with one or two FA (FA-FL, and FA-FL-FA), all made by chemo-enzymatic methods with excellent yield (95%+) and selectivity (100%) [21]. FA-FL-FA with two FA showed better endocytosis in both MDA-MB-231 (Caucasian) and MDA-MB-468 (African American, less FR) triple-negative breast cancer (TNBC) cell lines than FA-FL with a single targeting group. dPEG20, with precisely 20 repeat units with no polydispersity and two FA in each molecule, demonstrated the best uptake, in comparison with polydisperse PEGs with M_n_ = 1050 and 2000 g/mol. This was the first instance of using dPEG in FR-targeted PDCs. The uptake of FA-FL-PEG-FL-FA was monitored in vivo using a rat liver cancer model [22]. For intravenous delivery, tissue autofluorescence interfered with monitoring. In contrast, intra-arterial delivery led to accumulation in the tumor. FL is used extensively in cell culture studies but it is less than optimal for in vivo monitoring [14]. Therefore, we designed a new PDC platform based on a four-functional dPEG core to which drug and diagnostic molecules could be attached via enzyme-catalyzed Michael addition. The first compound tested was a bivalent folate-targeted PEGylated doxorubicin (DOX) serving as both a drug and an imaging agent, made by modular chemo-enzymatic processes (FA_2_-dPEG-DOX_2_) [23]. DOX is a widely used chemotherapeutic drug, which prohibits cell division by blocking the topoisomerase 2 enzyme [24]. It is also one of the most often chosen drugs in the synthesis of PDCs [25,26]. It fluoresces in red, enabling in vitro and in vivo tracking of drug release and distribution by fluorescent imaging techniques [27]. Our synthetic strategy is shown in Scheme 1. Exclusive *γ*-conjugation of FA was achieved using FA-SH made with a chemo-enzymatic method [28]. Flow cytometry analysis showed that, at 10 µM concentration, both free DOX and FA_2_-dPEG-DOX_2_ would be taken up by 99.9% of TNBC cells in 2 h. However, no cytotoxicity was found in the first 24 h. Slow cytotoxicity development led us to the conclusion that DOX was released slowly from the compound. Preliminary testing revealed that intra-tumoral injection of mice seemed to delay tumor growth more than intravenous delivery. Thus, this PDC showed great promise.

This paper discusses the synthesis of FA_2_-dPEG-DOX_2_ and the challenges associated with characterization of the compound.

## 2. Materials and Methods

Discrete poly(ethylene glycol) (dPEG_20_, FW = 882 g/mol, Ð = 1.00) was purchased from Quanta Biodesign Limited (Plain City, OH, USA). Doxorubicin hydrochloride (DOX.HCl, CAS 25316-40-9) was purchased from AvaChem Scientific (San Antonio, TX, USA). DL-α-tocopherol (Vitamin E, purity 97+%) was obtained from Alfa Aesar (Tewksbury, MA, USA). Thiol-functionalized folic acid (FA-SH) was synthesized as reported in [28]. *Candida antarctica* lipase B immobilized on acrylic resin (CALB, Novozyme 235), Vinyl acrylate (VA, <600 ppm MEHQ as inhibitor), diethanolamine (DEA, 99%), tetrahydrofuran (THF, ACS reagent grade), *n*-hexane (Hexane, ACS reagent grade), and anhydrous dimethyl sulfoxide (DMSO, ≥99.9%) were purchased from Sigma-Aldrich (Darmstadt, Germany) and used without further purification. Other solvents, such as anhydrous diethyl ether (95.8%, BHT free ACS Certified), methanol (ACS Certified), and acetone (ACS Certified), were obtained from Fisher Chemicals (Pittsburgh, PA, USA). Deuterated solvents, such as dimethyl sulfoxide (DMSO *d6*, purity 99.9%), chloroform (CDCl_3_, purity 99.8%), methylene chloride (CD_2_Cl_2_, purity 99.9%), and methanol (CD_3_OD, purity 99.8%) were purchased from Cambridge Isotope Laboratories (Tewksbury, MA, USA).

### 2.1. Synthesis

#### 2.1.1. Synthesis of Compound I: Acrylate-dPEG_20_-Acrylate

dPEG (1.4398 g, 0.0016 mol, 1 equivalent) was placed into a 25 mL round-bottom flask and dried under vacuum on a Schlenk line at 65 °C until bubble formation ceased. It was then cooled to room temperature and VA (0.3928 g, 0.0040 mol, 2.5 equivalents), CALB (0.1332 g @ 20 wt.% enzyme, 3 × 10^−4^ mol/L) and vitamin E (antioxidant) were added to the reaction mixture which was heated to 48 °C in an oil bath. After 4 h, the reaction mixture was diluted with 10 mL of dried THF. CALB was filtered over a Q5 filter paper and THF and VA were removed by a rotary evaporator under reduced pressure. The product was then dried in a vacuum oven. An amount of 1.3191 g (1.31 mmol) diacrylated product was obtained (82% yield).

#### 2.1.2. Synthesis of Compound II: (HO)_2_-dPEG_20_-(OH)_2_

Acrylate-dPEG_20_-acrylate (1.3191 g, 0.0013 mol, 1 equivalent), DEA (0.2782 g, 0.0026 mol, 2.02 equivalents) and 0.4 mL of DMSO were added to a 25 mL round-bottom flask and stirred at room temperature for 10 min. CALB (0.1089 g, 20 wt.% enzyme, 3 × 10^−4^ mol/L). One drop of vitamin E (antioxidant) was added to the reaction mixture which was then heated in an oil bath for 5 h at 50 °C. The reaction mixture was then taken out of the oil bath and diluted with 10 mL of THF. CALB was filtered over a Q5 filter paper and THF was removed using a rotary evaporator under reduced pressure. The product was then precipitated twice in 150 mL of hexane to remove excess DEA and DMSO, followed by drying of the product in a vacuum oven for 2 days. An amount of 1.0365 g (0.851 mmol) product was obtained (65% yield).

#### 2.1.3. Synthesis of Compound III: (Acr)_2_-dPEG-(Acr)_2_

(HO)_2_-dPEG_20_-(OH)_2_ (1.0365 g, 0.0009 mol, 1 equivalent) was mixed with VA (0.3427 g, 0.0035 mol, 4.10 equivalent) and 1.5 mL of CHCl_3_ and stirred at room temperature for 10 min. CALB (0.1133 g, 20 wt.% enzyme, 4 × 10^−4^ mol/L) and a drop of vitamin E (antioxidant) were added to the mixture which was kept in an oil bath for 5 h at 48 °C. Then the reaction mixture was diluted with 15 mL of THF. CALB was filtered over a Q5 filter paper and THF and VA were then removed by a rotary evaporator under reduced pressure. The product was then dried in a vacuum oven. An amount of 0.3290 g (0.229 mmol) (Acrylate)_2_-dPEG_20_-(Acrylate)_2_ was obtained (26% yield).

#### 2.1.4. Synthesis of Compound IV: FA_2_-dPEG-DOX_2_

Considering that the FA-SH used contained ~28 mol% of unreacted FA, FA-SH (0.3830 g, 0.0007 mol, 2.84 equivalents) was reacted with (Acrylate)_2_-dPEG_20_-(Acrylate)_2_ (0.3290 g, 0.0002 mol, 1 equivalent) using CALB (20 wt.% enzyme, 3 × 10^−4^ mol/L) in 1.4 mL of DMSO and a drop of vitamin E. The progress of the reaction was monitored by ^1^H-NMR. After 3 days, DOX (0.2659 g, 0.0005 mol, 2.13 equivalents) was first desalted using TEA and then added to the reaction mixture of the previous reaction. After running the reaction for 24 h at 65 °C, CALB was filtered out and the product was obtained by precipitation in 300 mL diethyl ether.

### 2.2. Characterization

#### Nuclear Magnetic Resonance (NMR) Spectroscopy

A Varian Mercury 300 MHz spectrometer was used to record the ^1^H-NMR spectra at 40 mg/mL concentration with the following parameters: 2 s relaxation time, 64 scans, and a 45° half-angle.

## 3. Results and Discussion

### 3.1. Synthesis of Acrylate-dPEG_20_-Acrylate (I)

**Figure 1:**
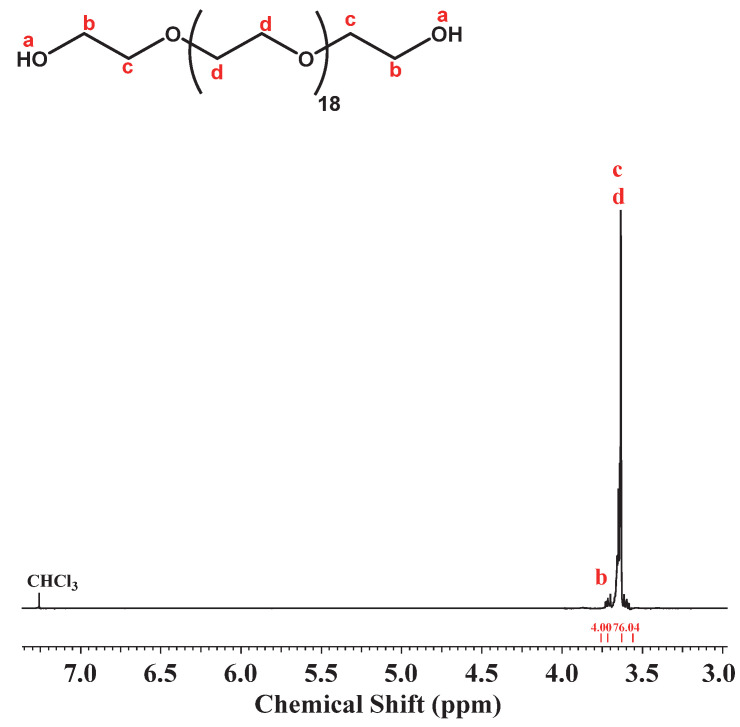
^1^H-NMR of dPEG_20_ ([δ 3.73 ppm ((b), s, 4H), 3.64 ((c, d), 76H)]).

Figure 1 shows the ^1^H-NMR of dPEG. The integral ratio of protons (c + d) with respect to proton (b) was 4.01:76.34, in excellent agreement with the theoretical 4:76 ratio.

**Figure 2:**
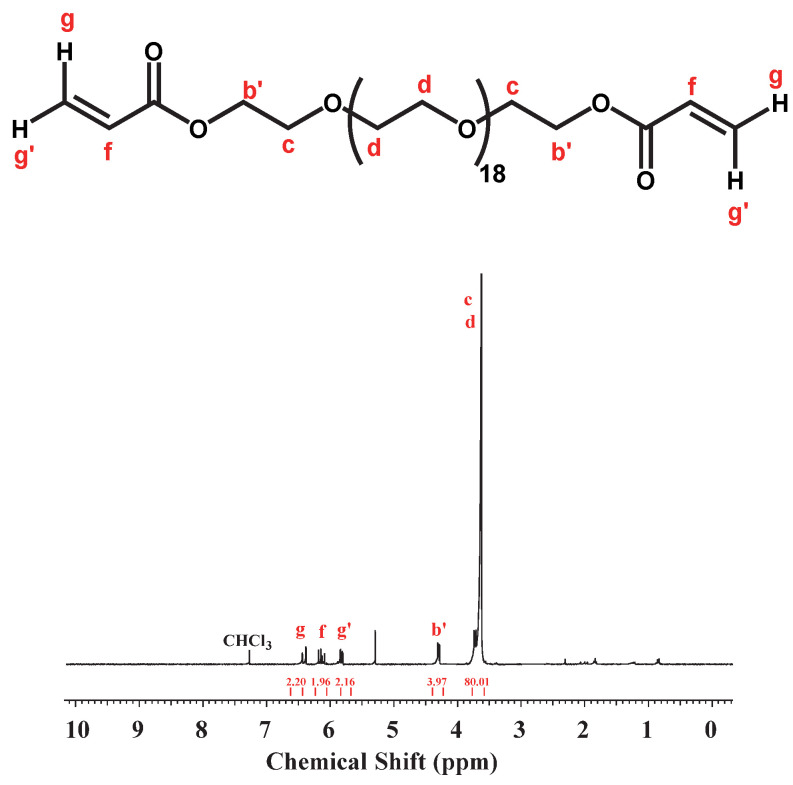
^1^H-NMR of the Acrylate-dPEG_20_-Acrylate in CDCl_3_ ([δ 6.50–6.38 ppm ((g), 2H), 6.21–6.07 ((f), 76H), 5.94–5.80 ((g’), 2H), 4.30 ((b’), 4H), 77–3.59 ((c,d), 80H)]).

dPEG was reacted with VA in bulk; no solvent was necessary because the liquified dPEG was miscible with VA. This reaction is irreversible because the vinyl alcohol product immediately tautomerizes into acetaldehyde that evaporates from the system. The ^1^H-NMR spectrum of the Acr-dPEG-Acr product after purification is shown in Figure 2. Resonance b shifted from 3.73 ppm to 4.30 ppm (b’). The integral ratio of the methylene (g, g’) and methine (f) protons of the acrylate group and proton (b’) relative to the reference protons of dPEG (c + d) was 2.20: 1.96: 2.16: 3.97: 80.01. This demonstrated successful transesterification between VA and dPEG and confirmed the structure of the diacrylate product.

### 3.2. Synthesis of (HO)_2_-dPEG_20_-(OH)_2_

**Figure 3:**
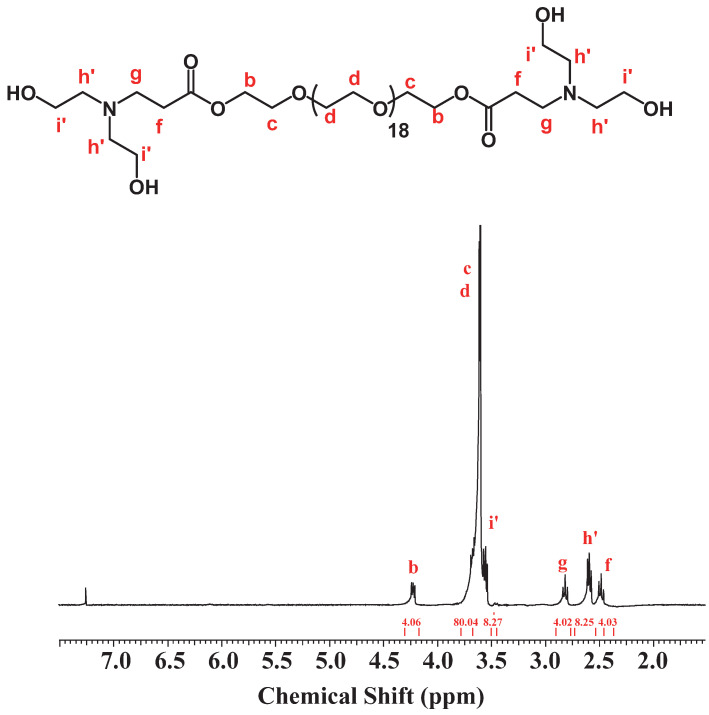
^1^H NMR spectrum of *(HO)_2_-dPEG_20_-(OH)_2_* ([δ 4.24 ((b), 4H), δ 3.77–3.59 ((c,d), 80H), δ 3.56 ((i’), 8H), δ 2.83 ((g), 4H), δ 2.59 ((h’), 8H), δ 2.48 ((f), 4H)]).

Acrylate-dPEG20-Acrylate was reacted with DEA in DMSO using CALB catalysis. Figure 3 shows the ^1^H-NMR of the product after purification and drying. Signal (i) of the DEA shifted from 3.42 to 3.56 ppm (i’), and signal (h) shifted from 2.54 ppm to 2.59 ppm (h’) after the reaction. No methylene and methine protons due to the acrylate were present. The integral ratio of proton (h’) and newly generated signals (g) and (f) with respect to the reference proton (b) was 8.25: 4.02: 4.03: 4.06 which demonstrated successful Michael addition between DEA and dPEG-diacrylate and confirmed the structure of the product.

### 3.3. Synthesis of (Acrylate)_2_-dPEG_20_-(Acrylate)_2_

**Figure 4:**
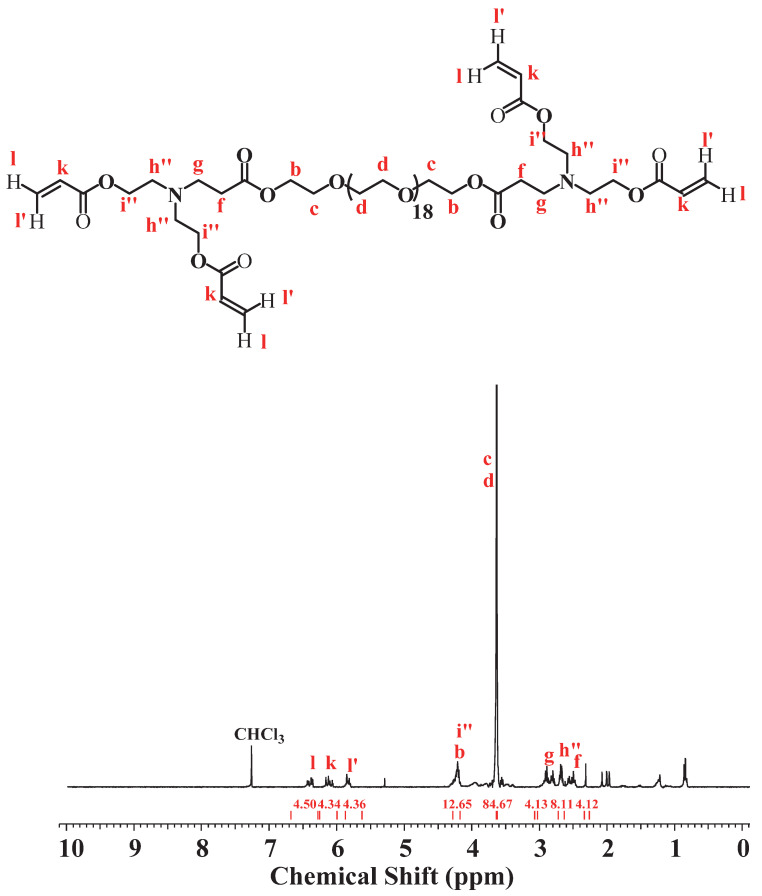
^1^H-NMR of the (Acrylate)_2_-dPEG_20_-(Acrylate)_2_ in CDCl_3_ ([δ 6.39 ppm ((l), 4H), δ 6.13 ppm ((k), 4H), δ 5.38 ppm ((l’), 4H), δ 4.21 ppm ((i”, b), 12H), δ 3.64 ppm ((c, d), 80H), δ 2.81 ppm ((g), 4H), δ 2.69 ppm ((h”), 8H), δ 2.54 ppm ((f), 4H)]).

(HO)_2_-dPEG_20_-(OH)_2_ was reacted with four equivalents of VA using CALB catalysis. As mentioned before, this transesterification reaction is irreversible. Figure 4 shows the ^1^H-NMR spectrum of the product after purification and drying. Signal (i’) moved from 3.56 ppm to 4.21 ppm (i”) and signal (h’) moved from 2.59 ppm to 2.69 ppm (h”) after the reaction.

New methylene and methine protons were generated at 6.39 ppm (l), 6.13 ppm (k) and δ 5.38 ppm (l’). The integral ratio of newly generated methylene and methine protons to the signal (i + b) was 4.50: 4.34: 4.36: 12.65. This demonstrated the successful transesterification reaction and confirmed the structure of the tetra-acrylated product. **This product (III) is the platform to which drugs and diagnostic agents can be attached to form PDCs. The first PDC made and tested was FA_2_-dPEG_20_-DOX_2_.**

### 3.4. Synthesis of FA_2_-dPEG_20_-DOX_2_

**Figure 5:**
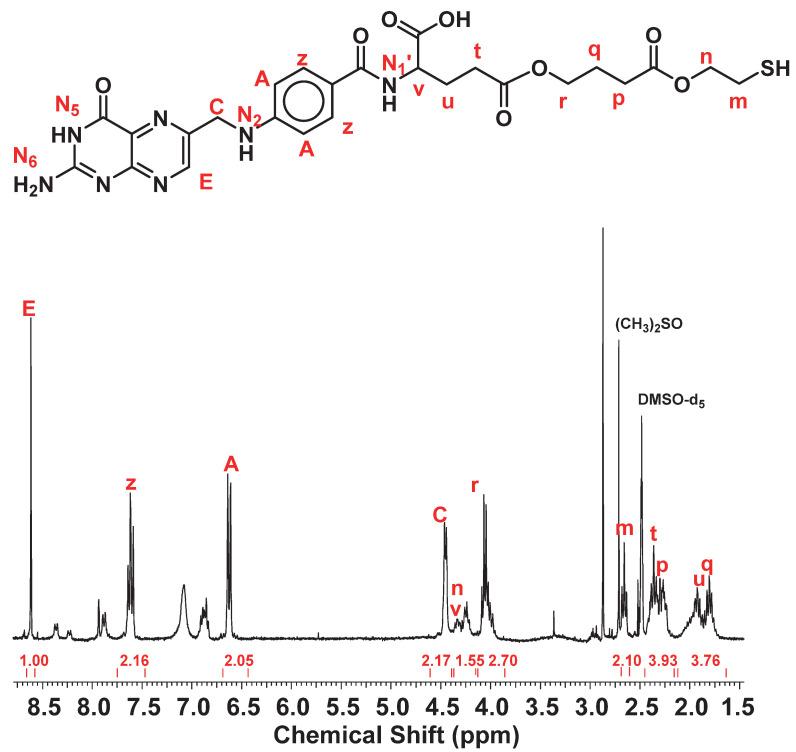
^1^H-NMR of the FA-SH in DMSO *d6* ([δ 8.60 ppm ((E), 1H), δ 7.60 ((z), 2H), δ 6.60 ((A), 2H), δ 4.40 ((C), 2H), δ 4.30-4.20 ((n, v), 3H), δ 3.95 ((r), 2H), δ 2.65 ((m), 2H), δ 2.25 ((t, p), 4H), 1.90 ((u), 2H), 1.50 ((q), 2H)]).

**Figure 6:**
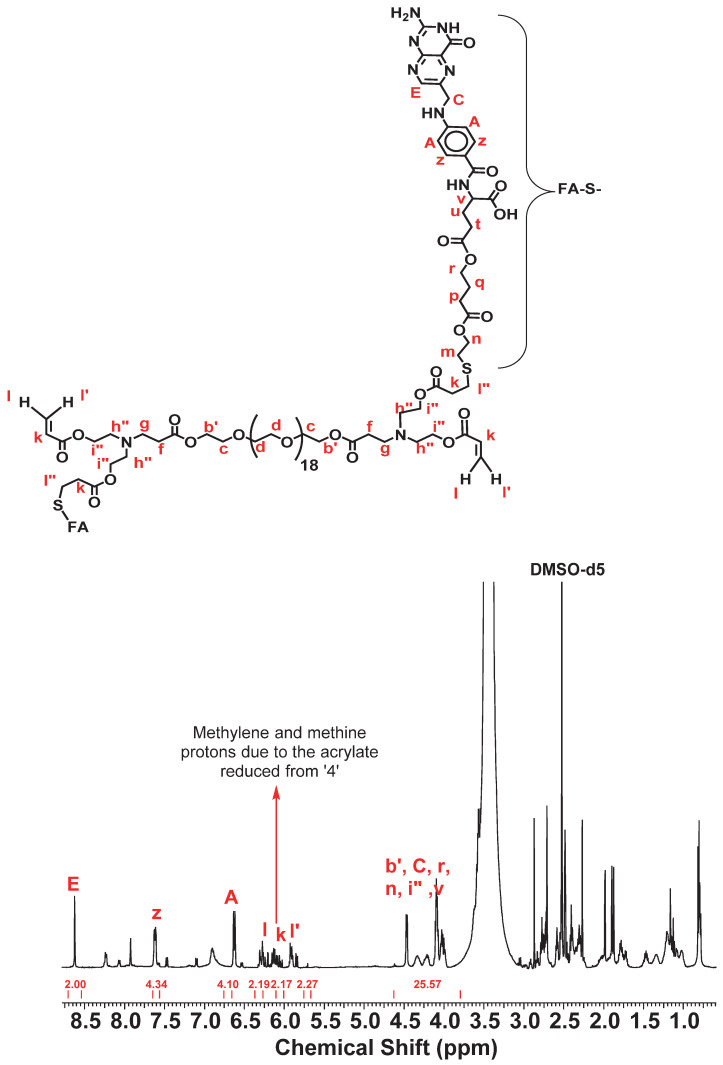
^1^H NMR of the raw sample of Michael addition between FA-SH and (Acrylate)_2_-dPEG_20_-(Acrylate)_2_ in DMSO *d6*.

FA-SH was attached to the (Acrylate)_2_-dPEG_20_-(Acrylate)_2_ by CALB-catalyzed Michael addition. The ^1^H NMR spectrum with assignments of FA-SH prepared as reported in [28] is shown in Figure 5. The ^1^H NMR spectrum of the product of the reaction can be seen in Figure 6.

Capital letters are used to mark the protons of the FA in Figure 6 as the spectra are quite complicated. The integral ratio of methylene protons (l) (6.39 ppm), (l’) (5.38 ppm) and methine protons (k) (6.13 ppm) with respect to the reference proton E of FA-SH was 2:2.20:2.28:2.18. Since the integral of the methylene and methine proton signals (l, l’, k) were reduced from 4 to 2, it was concluded that two of the acrylate groups reacted with FA-SH. The spectrum is very complicated with many overlaps, so only resonances assigned to protons l, k, l’, b, r, n, i, v, E, Z, A and C are identified in the spectrum.

**Figure 7:**
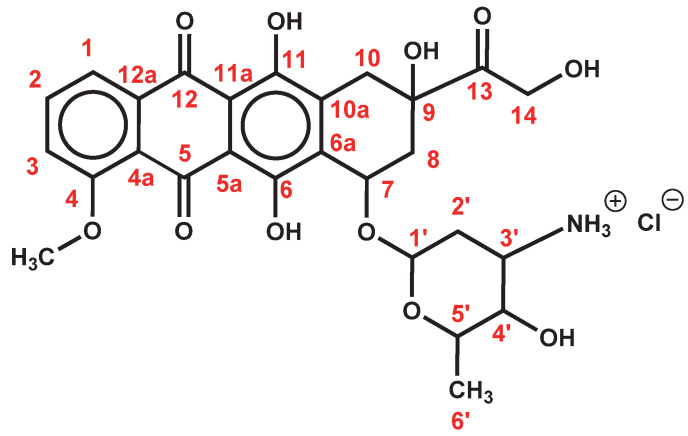
Structure of DOX.HCl: the NMR assignments listed in Table 1.

**Figure 8:**
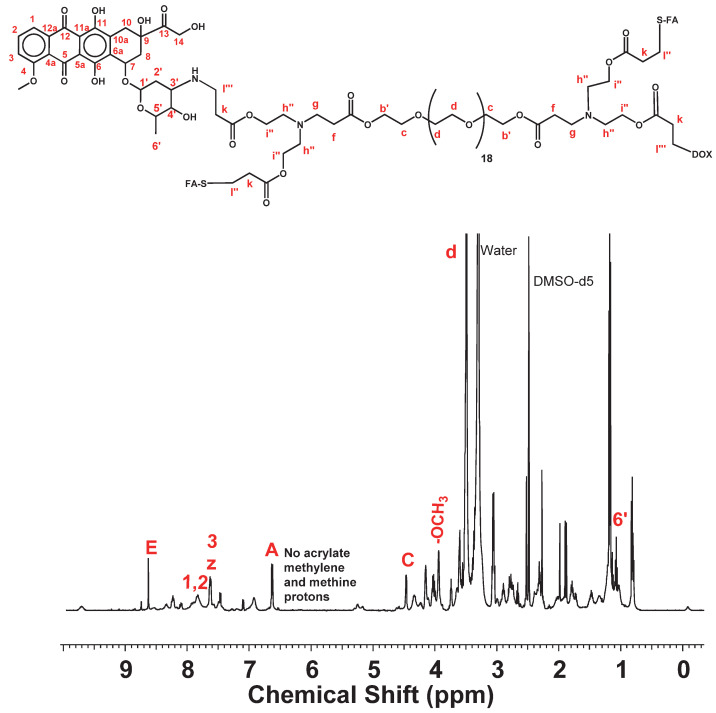
^1^H-NMR FA_2_-dPEG_20_-DOX_2_ in DMSO *d6*.

The structure of DOX.HCl is shown in Figure 7 (See the ^1^H NMR spectrum in Figure S1) Whereas the ^1^H NMR spectrum of FA_2_-dPEG_20_-DOX_2_ can be seen in Figure 8. Proper assignment of the NMR signals of DOX.HCl and some conjugates was published in 2017, correcting some errors in earlier publications [29]. When DOX is attached via amide bond formation from the *primary* amine after removal of the HCl, the signals associated with the protons 1′ through 6′ shift (see Table 1). Especially important is the proton in the position marked 3**′** at 3.37 ppm which was shown to shift to 3.94 ppm upon formation of an amide bond. However, this overlapped with the methyl protons of the methoxy group of DOX marked OCH_3_, also at 3.94 ppm, that remained in its original position.

The integral ratio of E from FA (see Figure 6), 6′ from DOX, and the dPEG main chain protons observed of 2:6:80, indicated that the desired FA_2_-dPEG_20_-DOX_2_ was obtained.

Some characteristic signals were able to be identified: E at 8.63 ppm from FA (see Figure 6), and 5′ at 4.16 ppm and 6′ (methyl protons) at 1.14 ppm from DOX. The ^13^C NMR was also crowded but shifts in some characteristic signals supported DOX conjugation: the 3′ signals shifted from 46.50 to 45.62 ppm, while the 2′ and 4′ signals shifted from 28.76 and 66.80 to 30.23 and 68.10 ppm, respectively (see Table 1 and Figure S2). The characteristic signal of γ-substituted FA was seen at 172.3 ppm, while the α carbonyl signal appeared at 173.7 ppm [28].

### 3.5. In Vitro and In Vivo Testing

**Figure 9:**
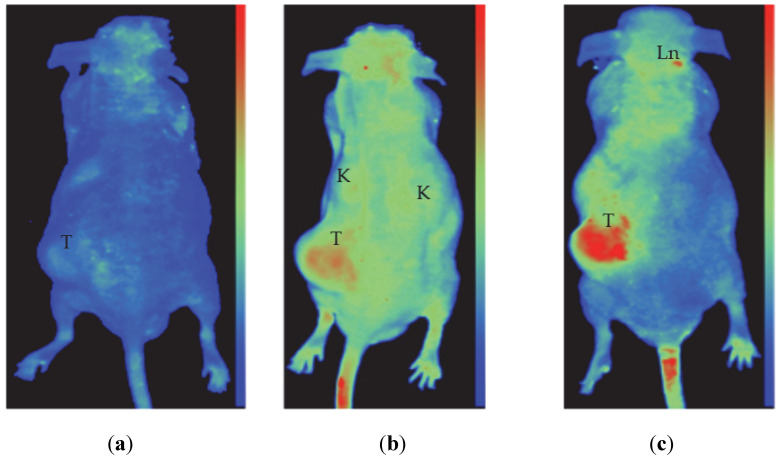
In vivo fluorescent images from the dorsal view of Foxn^Nu/Nu^ nude mice bearing LNCaP prostate xenograft tumors. Images before (**a**) and 24 h after IV (**b**) in one mouse, and after 24 h after intra-tumoral injection in another mouse (**c**). All images are standardized to identical light radiance minima and maxima in relative light intensity per pixel arbitrary units to allow for direct comparison. Fluorescence of the PDC is identified in the tumor (T) tail vein, kidneys (K), and capillary-rich head nuchal skin in (**b**), while a distinct fluorescent signal is observed in the tumor (T) and some lymph nodes of the neck (Ln) in the case of the intra-tumoral PDC injected animal (**c**). The slight autofluorescence, as seen in (**a**) before injection, is clearly different from the fluorescent signals of injected animals. Reproduced with permission from [23]. 2022, MDPI.

**Figure 10:**
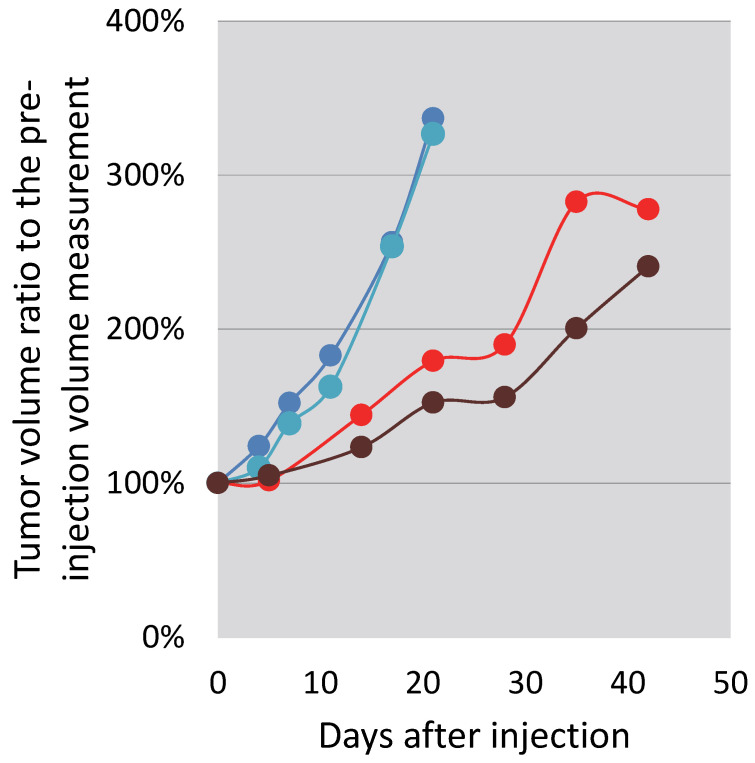
Follow-up of tumor volume increase in percentage of the initial tumor volume in a PC3-PSMA overexpressing cell line xenograft in two saline control (blue and turquoise) Nu/Nu nude mice, one injected intra-tumorally (brown) and one injected intravenously (red) with the PDC. Reproduced with permission from [23]. 2022, MDPI.

FA_2_-dPEG_20_-DOX_2_ was tested in vitro and in vivo in comparison with free DOX at the same concentration [22,23]. Free DOX already showed a cytotoxic effect after 24 h at 0.1 μM concentration, while no toxicity was observed with FA_2_-dPEG-DOX_2_. After 48 h treatment, the viability of the cells was reduced to 75% of the untreated control, even at the lowest (0.01 μM) concentration, and remained below the control level at all other concentrations applied. In comparison, the cytotoxicity of free DOX increased with increasing concentration, killing all cells at 100 μM. Preliminary testing in a live nude mouse model showed localization in an induced prostate cancer (PC3-PSCA-PSMA) tumor when delivered via intra-tumoral injection (Figure 9). The increase in the tumor volumes slowed down until Day 21 (see Figure 10). After this time point, the intravenously injected mouse tumor grew in a faster manner than the tumor of the intra-tumorally injected animal. Throughout the study, the intra-tumoral injection seemed to delay tumor growth more than the intravenous route of delivery. The results appear to indicate that DOX was released relatively slowly from the FA_2_-dPEG_20_-DOX_2_.

In summary, FA_2_-dPEG_20_-DOX_2_ seems to be a promising candidate as a folate-targeted cancer diagnostic and therapeutic agent, but more investigations are necessary in vitro and in vivo of this, and similar dPEG-based compounds, made by chemo-enzymatic synthesis.

We will continue research in the spirit of Andrzej’s legacy—another great scientist lost [2,30,31].

## 4. Conclusions

The chemo-enzymatic esterification and Michael addition reactions catalyzed by CALB are excellent selective reactions for the modification of PEGs. As was shown, a platform based on dPEG with four reactive acrylate groups was built with high selectivity. This is an excellent platform for the synthesis of a variety of polymer-based drug carriers. In the current investigation, we have shown that two folic acid groups and two doxorubicin groups could be attached to the platform using a CALB-catalyzed Michael addition reaction. We will expand this concept in the future for the synthesis of compounds with a variety of drugs and targeting agents.

## Figures and Tables

**Table 1:** NMR assignments from reference [29] in comparison with FA_2_-dPEG_20_-DOX_2_.

	DOX.HCl		DOC-NH-(C = O)-R		FA_2_-dPEG_20_-DOX_2_	
Group	H	C	H	C	H	C
OCH_3_ (4)	3.94	57.0	3.94	57.0	3.94	56.6
1′	5.25	99.7	5.18	100.9	5.18	-
2′	1.67; 1.87	28.6	(1.38); 1.79	30.2	1.79	30.2
3′	**3.37**	**46.9**	**3.94**	**45.3**	**3.94**	**45.9**
4′	3.61	66.6	3.37	68.6	3.35	69.7
4′-OH	5.46	-	4.70	-	4.46	-
5′	4.19	66.5	4.12	67.2	4.16	68.1
6′	1.14	17.2	1.19	17.6	1.17	19.6
			**FA-SH**		**FA_2_-dPEG_20_-DOX_2_**	
E			8.6		8.63	
z			7.6		7.63	
A			6.6		6.63	
C			**4.4**		**4.47**	
m			2.7			

## Data Availability

Raw data can be requested from the corresponding author.

## Supplementary Materials

The following supporting information can be downloaded at: https://www.mdpi.com/article/10.3390/polym14142900/s1, Figure S1: 1H NMR spectrum of DOX.HCl; Figure S2. ^13^C NMR spectrum of FA2-dPEG-DOX2
