# Supplementary material for: Multifunctional PEG Carrier by Chemoenzymatic Synthesis for Drug Delivery Systems: In Memory of Professor Andrzej Dworak
Source: Polymers (Basel). 2022 Jul 16;14(14):2900. doi: 10.3390/polym14142900 (PMC9320990; doi:10.3390/polym14142900)
Supplement: Supplementary file 1 [file polymers-14-02900-s001.zip › polymers-1782810-supplementary.pdf]

Multifunctional PEG Carrier by Chemoenzymatic Synthesis for Drug Delivery Systems: In Memory of Professor Andrzej Dworak

Judit E. Puskas [1,*], Gayatri Shrikhande [2], Eniko Krisch [1] and Kristof Molnar [1]

[1] Department of Food, Agricultural and Biological Engineering, College of Food, Agricultural, and Environmental Sciences, The Ohio State University, 222 FABE, 1680 Madison Avenue, Wooster, OH 44691, USA; molnarnekrisch.1@osu.edu (E.K.); molnar.182@osu.edu (K.M.)
[2] Dantari, Inc., 1290 Rancho Conejo Blvd, Suite 103, Thousand Oaks, CA 91320, USA; gayatri.shrikhande20@gmail.com
* Correspondence: puskas.19@osu.edu

Supporting Information

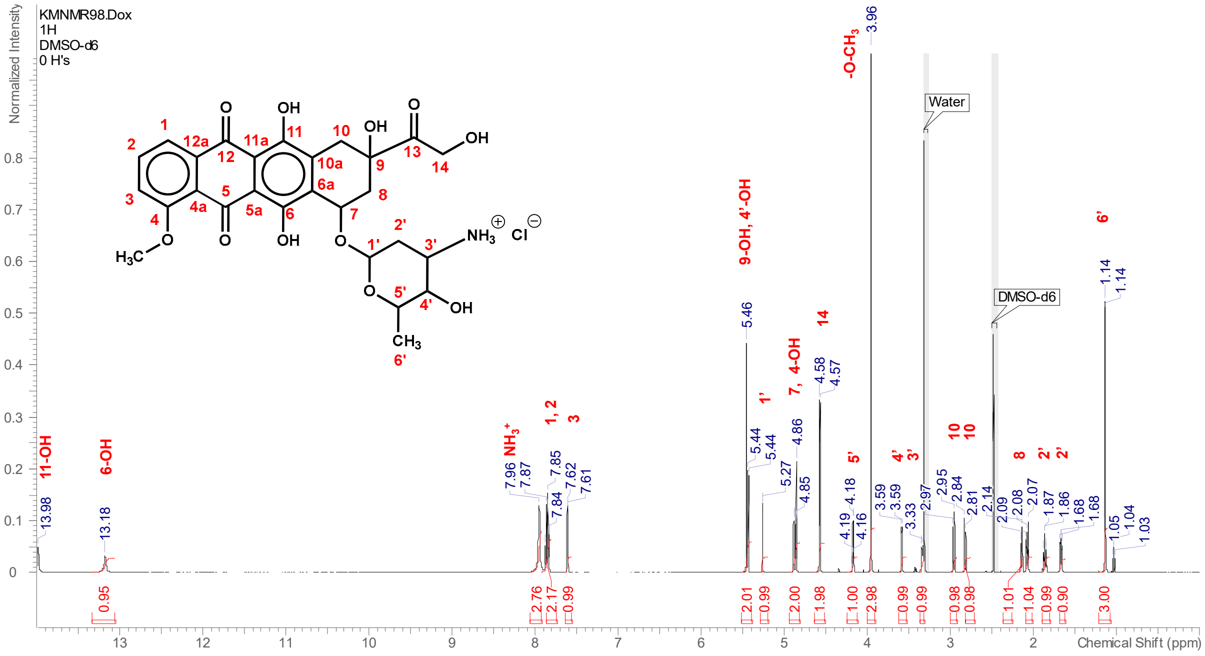

Figure S1. 1H NMR spectrum of DOX.HCl.

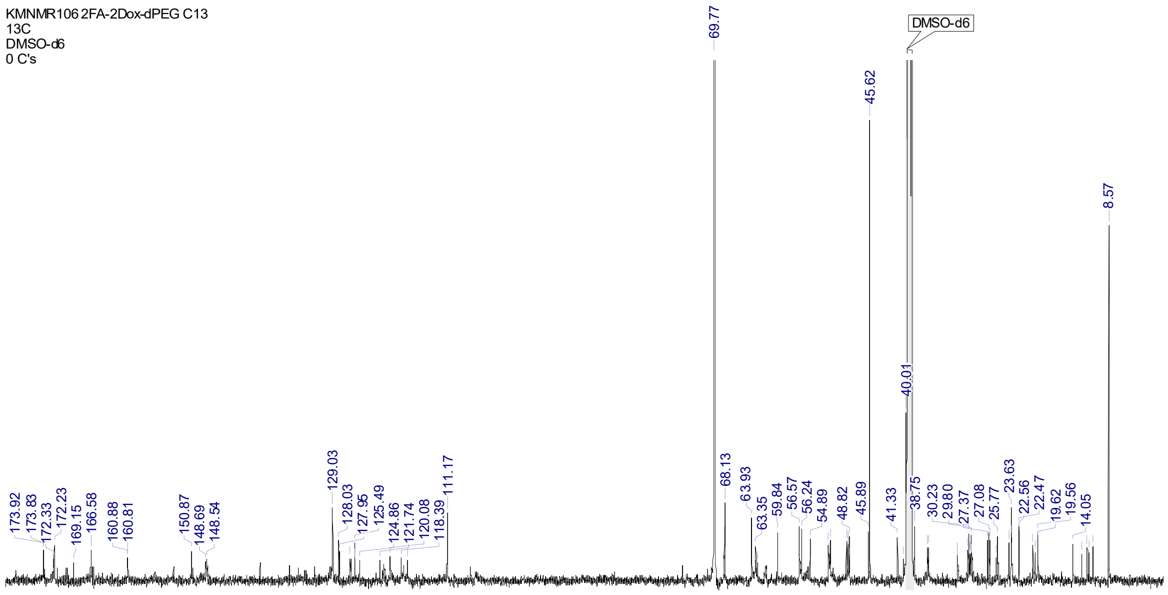

Figure S2. 13C NMR spectrum of FA_2-dPEG-DOX_2.
